# A comparison of thick-film microscopy, rapid diagnostic test, and polymerase chain reaction for accurate diagnosis of *Plasmodium falciparum* malaria

**DOI:** 10.1186/s12936-019-2711-4

**Published:** 2019-03-12

**Authors:** Kenji O. Mfuh, Olivia A. Achonduh-Atijegbe, Obase N. Bekindaka, Livo F. Esemu, Calixt D. Mbakop, Krupa Gandhi, Rose G. F. Leke, Diane W. Taylor, Vivek R. Nerurkar

**Affiliations:** 1 Department of Tropical Medicine, Medical Microbiology and Pharmacology, John A. Burns School of Medicine, University of Hawaii at Manoa, Honolulu, HI USA; 2 Pacific Center for Emerging Infectious Diseases Research, John A. Burns School of Medicine, University of Hawaii at Manoa, Honolulu, HI USA; 3 Biotechnology Center, University of Yaoundé I, Yaoundé, Cameroon; 4 National Medical Research Institute (IMPM), Yaoundé, Cameroon; 5 Biostatistics Core Facility Department of Complementary & Integrative Medicine, John A. Burns School of Medicine, University of Hawaii at Manoa, Honolulu, HI USA

**Keywords:** Malaria, Diagnosis, PCR, Microscopy, Clinical diagnosis

## Abstract

**Background:**

Accurate diagnosis of malaria is important for effective disease management and control. In Cameroon, presumptive clinical diagnosis, thick-film microscopy (TFM), and rapid diagnostic tests (RDT) are commonly used to diagnose cases of *Plasmodium falciparum* malaria. However, these methods lack sensitivity to detect low parasitaemia. Polymerase chain reaction (PCR), on the other hand, enhances the detection of sub-microscopic parasitaemia making it a much-needed tool for epidemiological surveys, mass screening, and the assessment of interventions for malaria elimination. Therefore, this study sought to determine the frequency of cases missed by traditional methods that are detected by PCR.

**Methods:**

Blood samples, collected from 551 febrile Cameroonian patients between February 2014 and February 2015, were tested for *P. falciparum* by microscopy, RDT and PCR. The hospital records of participants were reviewed to obtain data on the clinical diagnosis made by the health care worker.

**Results:**

The prevalence of malaria by microscopy, RDT and PCR was 31%, 45%, and 54%, respectively. However, of the 92% of participants diagnosed as having clinical cases of malaria by the health care worker, 38% were malaria-negative by PCR. PCR detected 23% and 12% more malaria infections than microscopy and RDT, respectively. A total of 128 (23%) individuals had sub-microscopic infections in the study population. The sensitivity of microscopy, RDT, and clinical diagnosis was 57%, 78% and 100%; the specificity was 99%, 94%, and 17%; the positive predictive values were 99%, 94%, and 59%; the negative predictive values were 66%, 78%, and 100%, respectively. Thus, 41% of the participants clinically diagnosed as having malaria had fever caused by other pathogens.

**Conclusions:**

Malaria diagnostic methods, such as TFM and RDT missed 12–23% of malaria cases detected by PCR. Therefore, traditional diagnostic approaches (TFM, RDT and clinical diagnosis) are not adequate when accurate epidemiological data are needed for monitoring malaria control and elimination interventions.

## Background

Malaria remains a major public health threat, particularly in sub-Saharan Africa, where about 191 million new infections and 395,000 deaths were reported in 2015 [1]. The World Health Organization (WHO) now recommends a confirmatory diagnosis of malaria using microscopy and/or RDT before initiation of treatment, partly influenced by the fear of the development of drug resistance and to enable the identification of malaria-negative patients, for which further investigations need to be sought for appropriate treatment [2]. Accurate diagnosis of malaria is thus vital for effective management and control of malaria while avoiding the wrong use of anti-malarial drugs. In most malaria-endemic countries, malaria is usually diagnosed by microscopy or rapid diagnostic tests (RDT) and clinical evidence.

The traditional practice by health care workers (HCW) in malaria-endemic countries has been to diagnose malaria based on a history of fever (clinical diagnosis) [3–6]. The specificity of clinical diagnosis of malaria is reduced by the overlap of malaria symptoms with other tropical diseases, such as typhoid fever, respiratory tract infections, bacterial disease and viral infections. The accuracy of clinical diagnosis may vary with the level of malaria endemicity, malaria transmission season and age group. Malaria microscopy is complex, which includes different species and blood stages of the *Plasmodium* parasite and requires a competent microscopist. Also, the presence of sub-microscopic parasitaemia greatly reduces the accuracy of malaria diagnosis by TFM. Unlike TFM, RDT detect malaria antigens, not malaria parasites, which gives them an added advantage in the ability to diagnose malaria in patients with low-grade parasitaemia below the detection limit of TFM [7, 8]. However, the specificity of the commonly used RDT that detects histidine rich protein–II (HRP-II) of *P. falciparum*, is limited when the parasite is cleared and antigens remain in circulation for about 28 days (false positive) [2].

As malaria-endemic countries move towards malaria elimination, there is a need for rapid and accurate diagnostic tools for malaria. Active monitoring of the performance of various diagnostic methods for malaria at the country level is necessary to guide policy on the diagnostic methods to use for malaria diagnosis and elimination. In this study, the performance of RDT, TFM and PCR in the diagnosis of malaria in Cameroon was compared in order to illustrate the number of cases missed by traditional methods (clinical diagnosis, RDT and TFM) of diagnosis.

## Methods

### Study area

This study was conducted in three Regions of Cameroon, Far North, Centre and North West with varied climatic conditions and altitudes (Table 1). In the Far North Region with seasonal malaria transmission the study was conducted in Maroua (10.5925°N, 14.3210°E) in October 2014. In the Central Region, which is holoendemic for malaria, the study was carried out in Nkolbisson (a neighbourhood in Yaoundé (3.8480°N, 11.5021°E) from February 2014 to April 2014. In the North West Region, which is holoendemic for malaria, the study was conducted in Bamenda (5.9631°N, 10.1591°E) in February 2015.

**Table 1:** General characteristics of study population, study sites and environmental factors

Characteristics	Study sites			Total, n (%)
	Maroua, n (%)	Nkolbisson, n (%)	Bamenda, n (%)	
*Gender*				
Male	64 (52)	168 (53)	33 (29)	265 (48)
Female	60 (48)	147 (47)	79 (71)	286 (52)
Total	124	315	112	551
*Age group (years)*				
0–5	81 (65)	180 (57)	34 (30)	295 (54)
6–10	15 (12)	86 (27)	5 (4)	106 (19)
11–16	9 (7)	49 (16)	4 (4)	62 (11)
≥ 17	19 (15)	0 (0)	69 (61)	88 (16)

Environmental factors [10]	Maroua	Nkolbisson	Bamenda	
Climate	Sahel	Tropical	Tropical	
Average annual temperature (°C)	28.3	23.7	21.5	
Average annual rainfall (mm)	794	1643	2145	
Elevation (m)	384	750	1614	
Weather condition at time of specimen collection	End of rainy season	Rainy season	Dry season	

### Study design

A cross-sectional study was conducted in selected health facilities in Maroua, Nkolbisson, and Bamenda. Inclusion criteria were age > 6 months and axillary temperature >37.5 °C at the time of recruitment or fever within 24 h preceding recruitment. A written informed consent was obtained from all study participants ≥ 18 years of age. Parents or legal guardians of children < 18 years gave a written informed consent on behalf of their children.

### Ethical considerations

Ethical approvals were obtained from the Committee on Human Subjects of the University of Hawaii (protocol number CHS 21724) and the National Research Ethics Committee of the Ministry of Public Health, Cameroon (protocol number 2014/04/442/CE/CNERSH/SP). Administrative approvals were obtained from the Ministry of Public Health, Cameroon and the Directors of the participating facilities.

### Study procedures

An easy-to-read questionnaire was used for the collection of demographic and clinical data. After obtaining informed consent, the research or clinic staff took axillary temperatures and recorded the reported signs and symptoms. Venous blood, 2–5 mL, was collected from each participant, dispensed into ethylenediaminetetraacetic acid (EDTA) tubes and stored in cold boxes until transported to the research laboratory where they were stored at 4–8 °C. TFM and RDT for malaria were conducted for all participants, and both results were presented to the consulting health care worker (HCW). At the end of the hospital visit, an exit survey was conducted to obtain information on (1) the clinical diagnosis made by the HCW, and (2) determine if anti-malarial drugs had been prescribed. This information was used to determine how frequent anti-malarial drugs were used to treat malaria-negative patients. When exit survey information was not available, the hospital record was consulted for the information.

### Laboratory investigations

#### Malaria TFM

Thick blood films were prepared and stained using 10% Giemsa for 15 min. A slide was considered positive if at least one asexual blood-stage *P. falciparum* parasite was identified. Parasitaemia was determined by counting the number of parasites per 200 white blood cells and assuming that each subject had 8000 white blood cells/μL of blood. Two readings were conducted for each slide and discrepancies greater than 10% were resolved by a third reading by an independent trained microscopist. The slides for this study were read by well-trained and experienced scientists who have worked in malaria-research for a number of years.

#### Malaria RDT

Approximately 5 μL of blood was used to diagnose malaria using the Ag Pf/Pan malaria RDT kit (Standard Diagnostic Inc., South Korea), following the manufacturer’s instructions. This RDT is a qualitative immunochromatographic test that detects *P. falciparum* HRP-II and *Plasmodium* lactate dehydrogenase, which is a glycolytic enzyme common to *P. falciparum, Plasmodium ovale, Plasmodium vivax* and *Plasmodium malariae* asexual-stage parasites.

#### Malaria PCR

DNA was extracted from 200 μL of whole blood using the mini-prep spin-column technique (Macherey-Nagel, Germany) following the manufacturer’s instructions. Detection of malaria parasite DNA was based on nested PCR amplification of the 18 s rRNA gene in a reaction that used 2 μL of the extracted DNA, 10 μL of GoTaq polymerase and master mixes (Promega, USA), 0.25 μM each of upstream and downstream primers, and 6 μL of nuclease free water in a total reaction volume of 20 μL. The first PCR encompassed genus-specific primers and the second nested PCR run encompassed the species-specific primers for *P. falciparum, a*s previously described [9]. The presence of a characterizing band of ~ 205-bp for *P. falciparum* visualized on a UV transilluminator after electrophoresis on a 2% agarose gel stained with ethidium bromide.

### Statistical analysis

Data were entered into Microsoft Office Excel and analysed using StatPlus 5.9.80 (AnalystSoft Inc., Walnut, CA) and Prism 6.0 (Graphpad Software, San Diego, CA) for descriptive statistics. Diagnostic test performance for clinical diagnosis, TFM and RDT for the diagnosis of malaria was analysed using MedCalc 16.8 (Ostend, Belgium). Descriptive statistics are represented as frequencies and medians. Sensitivity, specificity, positive and negative predictive values, accuracy and percentage of agreement (*kappa* value) were calculated with confidence intervals by age groups. Multivariable logistic regression was conducted to identify correct diagnosis comparing other test methods (TFM and RDT) to PCR. *P *< 0.05 was considered statistically significant. All the other analyses were conducted using SAS version 9.4 (SAS Institute Inc., Cary, NC). The figure was generated using R version 3.4.2.

## Results

### General characteristics of the study population and study site environmental factors

Of the 551 febrile patients recruited from the selected health care facilities in Cameroon, 57% resided in Nkolbisson; 23% in Maroua and, 20% in Bamenda. The overall distribution of males and females was 48% and 52%, respectively. The majority of the study participants were in the age-group of 0–5 years, with 81 (65%) in this age group in Maroua, and 180 (57%) in Nkolbisson. In Bamenda however, 69 (61%) of the study participants were over 17 years of age (Table 1).

### Proportion of diagnosed malaria cases by various diagnostic methods

The overall proportion of participants diagnosed with malaria by TFM, RDT, and PCR in the cohort was 31%, 45%, and 54%, respectively. However, 92% (507/551) of the participants were diagnosed with clinical malaria (temperature range 36.5 to 39.7 °C), of which 41% (209/551) were negative for malaria by PCR (Table 2). All the participants (507) who were diagnosed with clinical malaria were prescribed anti-malarial drugs by the HCW. The prevalence of malaria by PCR in Maroua, Nkolbisson, and Bamenda was 57%, 70% and 5%, respectively. Therefore, a proportion of malaria cases were missed by TFM and RDT. Unfortunately, 41% of patients were falsely diagnosed as being positive for malaria by HCW.

**Table 2:** Malaria prevalence stratified by test method and study site

Test method	Study sites			Total (n = 551)
	Maroua (n = 123)	Nkolbisson (n = 315)	Bamenda (n = 112)	
	MP+ (%)	MP+ (%)	MP+ (%)	MP+ (%)
TFM	32 (26)	135 (43)	5 (4)	172 (31)
RDT	67 (54)	175 (55)	7 (6)	249 (45)
PCR	71 (57)	221 (70)	6 (5)	298 (54)
Clinical diagnosis	120 (97)	301 (96)	86 (77)	507 (92)

### Sub-microscopic infections detected by RDT and PCR

Sub-microscopic *P. falciparum* infections were defined as i) positive by PCR but negative by microscopy, ii) positive by PCR but negative by RDT, and iii) positive by RDT but negative by TFM. A total of 128 (23%) febrile patients were positive for *P. falciparum* by PCR, but negative by TFM (Table 3). The lowest parasitaemia detected by TFM was 120 parasites/μL of blood. Meanwhile, 65 (12%) febrile patients were positive for *P. falciparum* by PCR and negative by RDT. However, 84 (15%) patients were positive for malaria by RDT but negative by PCR. This shows that, 23% of malaria cases were missed by TFM while RDT detected 15% more malaria infections compared to TFM but failed to detect 12% detected by PCR.

**Table 3:** Prevalence of sub-microscopic *P. falciparum* infection by age-group

Age-group^a^ (n)	Median parasites/μL (range: Q1–Q3)	Test characteristic		
		PCR (+), TFM (−) n (%)	PCR (+), RDT (−) n (%)	RDT (+), TFM (−) n (%)
0–5 (295)	12,405 (1500–300,000)	72 (28)	45 (17)	37 (14)
6–10 (106)	15,200 (3200–300,000)	34 (32)	12 (11)	27 (25)
11–16 (62)	8640 (3360–75,200)	16 (26)	5 (8)	13 (21)
≥ 17 (88)	721 (461–852)	6 (7)	3 (3)	7 (8)
Total (n = 551)		128 (23)	65 (12)	84 (15)

PCR (+), TFM (−) = PCR positive but TFM negative

PCR (+), RDT (−) = PCR positive but RDT negative

RDT (+), TFM (−) = RDT positive but TFM negative

Q1–Q3: 25% to 75% interquartile range

*TFM* thick film microscopy, *RDT* rapid diagnostic test, *PCR* polymerase chain reaction

^a^In years

### Proportion of diagnosed malaria cases by test method in different age group

**Fig. 1:**
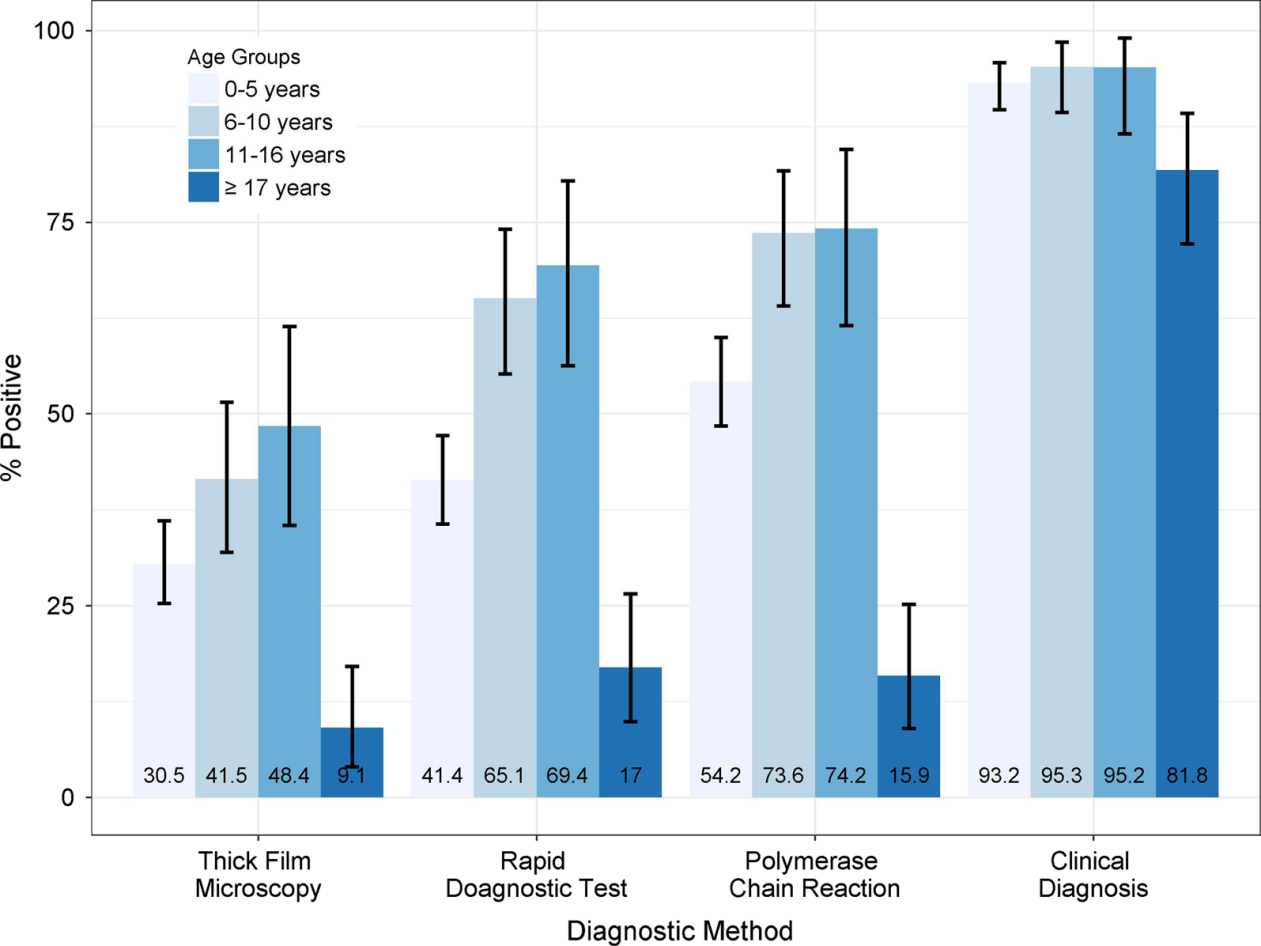
Age-specific detection of malaria by diagnostic method

Across the different age-groups, RDT and PCR detected more malaria infections as compared to TFM (Fig. 1). Moreover, the age-specific detection of malaria by RDT was closer to that of PCR. However, the rate of clinical diagnosis of malaria was high irrespective of the age-group. There was no significant difference in accurately diagnosing malaria by RDT (95% CI 0.52–2.57; p = 0.23) and TFM (95% CI 0.77–2.96; p = 0.73) between those aged ≤ 5 years and > 5 years.

Also, across the different age-groups, the sensitivity and specificity of TFM and RDT were similar (Table 4). This means that across different age-groups, RDT was almost as good as PCR, but still missed 12% of malaria cases. Also, both RDT and TFM work well across different age-groups.

**Table 4:** Performance of TFM and RDT across age–groups with PCR as reference method

Age group (years)	Sensitivity TFM (95% CI)	Specificity TFM (95% CI)	Sensitivity RDT (95% CI)	Specificity RDT (95% CI)
0–5	55% (45–63)	98% (95–99)	72% (64–79)	95% (90–98)
6–10	56% (45–68)	100% (88–100)	85% (75–92)	89% (72–98)
11–16	65% (50–79)	100% (79–100)	89% (76–96)	87% (62–98)
≥ 17	57% (29–82)	100% (95–100)	79% (49–95)	95% (87–98)

*TFM* thick film microscopy, *RDT* rapid diagnostic test, *PCR* polymerase chain reaction, *CI* confidence interval

### Comparison of diagnostic accuracy of clinical diagnosis, TFM and RDT vs. PCR

Using PCR as reference standard, 298 participants were positive for malaria while 253 participants were negative for malaria (Table 5). RDT correctly identified 234 (79%) infections (true positive), microscopy correctly identified 170 (57%) infections; whereas clinical diagnosis identified all 298 (100%) infections. However, there were 209 (83%) false positive clinical diagnosis, 15 (6%) by RDT and 2 (0.8%) by TFM. The sensitivity of TFM, RDT, and clinical diagnosis was 57%, 78%, and 100%, respectively; the specificity was 99%, 94%, and 17%, respectively; the positive predictive value was 99%, 94%, and 59%, respectively, and negative predictive value was 66%, 78%, and 100%, respectively. The clinical diagnosis had a “poor” agreement (kappa 0.18), malaria RDT had a “good” agreement (kappa 0.71), and malaria microscopy had a “moderate” agreement (kappa 0.54) when compared to PCR. In general, the accuracy of clinical diagnosis was 62%, RDT 85%, and TFM 76%. However, the error rate of clinical diagnosis was 34%, RDT was 14%, and TFM was 23% (Table 5).

**Table 5:** Diagnostic test performance of clinical diagnosis, RDT, and TFM in the diagnosis of malaria with PCR as reference method

Test characteristic	Clinical diagnosis	RDT	TFM
TP (PCR = 298)	298	234	170
FP (PCR negative)	209	15	2
TN (PCR = 253)	44	237	251
FN (PCR positive)	0	65	128
Sensitivity [95% CI]	100% [99–100]	78% [73–82]	57% [51–63]
Specificity [95% CI]	17% [13–23]	94% [90–97]	99% [97–99]
PPV [95% CI]	59% [54–63]	94% [90–96]	99% [96–99]
NPP [95% CI]	100% [92–100]	78% [73–83]	66% [61–71]
Accuracy [95% CI]	62% [58–66]	85% [82–88]	76% [73–80]
Kappa value [95% CI]	0.18 [0.14–0.24]	0.71 [0.65–0.77]	0.54 [0.48–0.60]
Misclassification rate (%)	34	14	23

*TP* true positive, *FP* false positive, *TN* true negative, *FN* false negative, *PPV* positive predictive value, *NPV* negative predictive value

## Discussion

Accurate and prompt diagnosis of malaria is the only way to effectively treat, manage and eventually eliminate the disease. This study was conducted to determine the proportion of malaria cases missed by conventional malaria diagnostic methods, namely, TFM, RDT, clinical diagnosis, but detected by PCR.

In this study, TFM missed 23% of PCR-positive malaria infections. In a previous meta-analysis based on data from 42 studies, microscopy missed about 50% of PCR-positive malaria infections [11]. Also, in a large epidemiological study in Cambodia a significant proportion of microscopy-negative samples were detected by PCR (289/7491; 3.85%) [12]. False-negative microscopy results are known to increase as parasite density decreases [13]. Moreover, the detection threshold of Giemsa-stained TFM varies considerably between 50–500 parasites/μl of blood. However, TFM predicted the presence of malaria parasite in 99% of the study participants making TFM a good “rule in” test for malaria. This means that a positive TFM result for malaria can be trusted; meanwhile, a negative result does not exclude the presence of malaria infection.

Malaria RDT missed 12% of PCR-positive malaria infections. However, the accuracy of malaria RDT was good as compared to PCR. The sensitivity and specificity of malaria RDT in this study was 78% and 94% respectively, which is consistent with a recent study in Kenya [14] that evaluated the same RDT. Several factors have been demonstrated to affect the sensitivity of RDTs based on detection of HRP-II, including an inherent limitation of the device, mutation or deletion of the gene encoding the HRP-II, and storage conditions [13, 15]. Interestingly, RDT detected 15% of malaria cases that were missed by TFM. This is because RDT detects antigens, not parasites, which gives it an added advantage over microscopy in its ability to diagnose malaria in patients with low parasitaemia below the detection threshold of microscopy [7, 8]. However, there is the possibility of false positive RDT results when the malaria parasite is cleared, and antigens remain in circulation. In the present study, malaria RDT could predict the presence of malaria parasite in 94% of the study participants making it a good “rule in” test for malaria. Therefore, in the absence of PCR, malaria RDT can be used to improve the quality of care by ensuring appropriate treatment of confirmed malaria cases while avoiding indiscriminate administration of anti-malarial drugs for malaria-negative patients. However, because a substantial proportion of infections were missed by RDT, it is therefore not sufficiently sensitive for mass screening programmes as recommended by the WHO [16].

This study found high rates of clinical diagnosis and overtreatment of malaria, in all three Cameroonian study sites, which is consistent with previous studies [17–20]. Even though the sensitivity of clinical diagnosis of malaria was high, the overall accuracy of clinical diagnosis was poor with 41% of malaria-negative patients erroneously treated for malaria. Moreover, clinical diagnosis of malaria could predict the presence of malaria parasite in only 59% of study participants. Clearly, many patients that were treated for malaria had other causes of fever. Therefore, clinical diagnosis of malaria cannot be relied upon as a “rule in” test for malaria due to overlapping malaria symptoms with other tropical febrile illnesses.

Results of this study also provide information on the prevalence of malaria in three Regions of Cameroon. The prevalence of malaria was high in Nkolbisson and Maroua, but low in Bamenda. These three regions are characterized by different climatic variables (Table 1), which have been shown to affect the prevalence of malaria in a given region [21–25]. A previous study in Tanzania reported malaria prevalence proportions of 79–90%, 27–46% and 8–16% in low, intermediate and high altitudes, respectively [26]. Results of this study thus provide information on the prevalence of malaria in three climatically different regions of Cameroon, which is important to guide malaria control interventions.

This study has a limitation in that quantitative PCR was not conducted in order to quantify parasitaemia. Thus, it was not possible to stratify the infections that were missed by TFM and RDT by level of parasite density in order to determine the variation in sensitivity across parasite density levels.

## Conclusions

Results of this study suggest that the conventional diagnostic methods for malaria (TFM, RDT,) are not adequate when accurate epidemiological data are needed to monitor malaria control and elimination interventions. PCR permitted the detection of 23% of malaria cases missed by TFM and RDT further, confirming it as a valuable tool for epidemiological surveys, mass screening, and the assessment of interventions for malaria elimination. Therefore, the development and standardization of a rapid and sensitive molecular-based test capable of detecting sub-microscopic malaria infection are warranted for the global elimination of malaria. Furthermore, continual training and proficiency testing should be instituted for laboratory technicians on malaria microscopy and post-market surveillance to assure the quality of malaria RDT since high-performance microscopy and quality assured RDTs will suffice for the clinical management of patients with suspected malaria.

## Abbreviations

HRP-2: histidine-rich protein-II
nPCR: nested PCR
NLR: negative likelihood ratio
NPV: negative predictive value
PCR: polymerase chain reaction
PLR: positive likelihood ratio
PPV: positive predictive value
RDT: rapid diagnostic test
HCW: Health Care Workers
TFM: thick film microscopy
